# Enteric neurons show a primary cilium

**DOI:** 10.1111/j.1582-4934.2012.01657.x

**Published:** 2012-12-04

**Authors:** Mª José Luesma, Irene Cantarero, Tomás Castiella, Mario Soriano, José Manuel Garcia–Verdugo, Concepción Junquera

**Affiliations:** a Department of Human Anatomy and Histology, Faculty of Medicine, University of ZaragozaZaragoza, Spain; b Aragon Health Research Institute (IIS Aragón), Scientific Research Centre of AragónZaragoza, Spain; c Príncipe Felipe Research CentreCell Biology Laboratory, Valencia, Spain

**Keywords:** primary cilia, neuronal cilia, enteric ganglia, enteric nervous system, ultrastructure

## Abstract

The primary cilium is a non-motile cilium whose structure is 9+0. It is involved in co-ordinating cellular signal transduction pathways, developmental processes and tissue homeostasis. Defects in the structure or function of the primary cilium underlie numerous human diseases, collectively termed ciliopathies. The presence of single cilia in the central nervous system (CNS) is well documented, including some choroid plexus cells, neural stem cells, neurons and astrocytes, but the presence of primary cilia in differentiated neurons of the enteric nervous system (ENS) has not yet been described in mammals to the best of our knowledge. The enteric nervous system closely resembles the central nervous system. In fact, the ultrastructure of the ENS is more similar to the CNS ultrastructure than to the rest of the peripheral nervous system. This research work describes for the first time the ultrastructural characteristics of the single cilium in neurons of rat duodenum myenteric plexus, and reviews the cilium function in the CNS to propose the possible role of cilia in the ENS cells.

## Introduction

Primary cilia are slender microtubule-based organelles (9+0) that usually exist in a single copy on the surface of several cell types [1, 2]. Initially it was thought that the primary cilium in non-mitotic cells had a non-functional vestigial role [3, 4]. Now primary cilia can be viewed as sensory cellular antennae that could co-ordinate some cellular signalling pathways [5]. A number of specific receptors, ion channels, transporters and downstream signalling proteins have been localized to primary cilia, thus allowing the cilium to detect and transduce extracellular signals to the inside of the cell [6]. In this way the primary cilium functions as a unique cellular site for mechano-, chemo- and osmo-sensation to regulate cellular processes during development and in tissue homeostasis [7].

The structural proteins and the signalling proteins required for cilia function are synthesized in the cell body and transported into the cilium by a mechanism known as intraflagellar transport (IFT) [8–10]. IFT is required for the formation and maintenance of all mammalian cilia, and defective IFT is associated with severe diseases and developmental defects collectively called ‘Ciliopathies’ [11–15].

The presence of primary cilia in the nervous system was first detected in neurons in sympathetic ganglia of amphibians and reptilia over 50 years ago [16]. In the mammalian central nervous system, neuronal cilia were recognized as common ultrastructural features of granule cells in rat dentate gyrus [3], major neuron types in rat cerebellum [17, 18], immature rat supraoptic nucleus [19], guinea pig cerebellum, hypothalamus and neocortex [20], some types of retinal neurones in cat and rabbit [21], hamster paraventricular hypothalamic nucleus [22], human neocortex [23] and neurons of rat striatum [24]. Primary cilia have also been described in Schwann cells in the autonomic nervous system [25].

Specific proteins have been shown to selectively localize to neuronal cilia and can serve as markers by immunolabelling [4, 26]; cilia express a complement of proteins distinct from other neuronal compartments. It has also been recently shown that somatostatin sst3 receptors [27, 28], serotonin 5-HT receptors [29] and dopamine receptors 1 (D1) localize to cilia in mouse and rat central neurons [30–32].

Several authors have also reported the presence of a single cilium as an ultrastructural characteristic of precursor cells, such as astrocyte-like neural precursors in neurogenesis in the hippocampal region [33] or neural progenitor cells in the subventricular zone of adult mouse [34–37]. Different studies have shown that the primary cilium concentrates components of Shh signalling, sonic hedgehog-triggered proliferogenic signalling from the primary cilia on granule cell progenitors in the adult dentate subgranular zone to maintain a pool of new dentate granule cells [38–41].

Two recent reports (on medulloblastoma and basal-cell carcinoma) confirm the relationship between primary cilia and Hedgehog-dependent cancers [42, 43]. Thus, at present, new lines of research have branched off to investigate the role of primary cilia in neuronal signalling, adult neurogenesis and brain tumour formation.

The presence of primary cilia in the enteric nervous system (ENS) has not yet been described in mammals to the best of our knowledge. This study describes the location and ultrastructural characteristics of the primary cilia in neurons in the myenteric plexus of the rat duodenum. This first description will serve as a basis for further research.

## Material and methods

### Animal use

Four adult Wistar rats, 3 months old, weighing between 190 and 210 g. (from Jackson Laboratory, Bar Harbor, ME, USA) were used in accordance with institutional guidelines (Ethics Advisory Commission for Animal Experimentation, PI 36/10). Each animal had *ad libitum* access to food and water and was fed on a complete and balanced standard laboratory diet. The animals were housed in temperature controlled rooms (20 ± 1°C) and natural light. All the rats were anaesthetized, and perfused intracardially with 2.5% glutaraldehyde and 2% paraformaldehyde following a modified Karnovsky's protocol [44].

### Transmission electron microscopy (TEM)

After the intestine extraction, the mucosa from the duodenum samples is eliminated, being the final size of about 1–1.5 mm^3^ (corresponding to the muscular coat). Afterwards, they were washed in phosphate buffer and fixed with 2.5% glutaraldehyde and 2% paraformaldehyde (in PB 0.1 M) overnight at room temperature, washed in 0.1 M phosphate buffer for 5 min., post-fixed with 2% osmium (in PB 0.1 M), rinsed, dehydrated in graded acetones (30%, 50%, 70% with 2% uranyl acetate, 90%, 100%), cleared in propylene oxide and embedded in araldite (Durcupan, Fluka AG, Buchs SG, Switzerland). The samples were oriented in a perpendicular plane using flat moulds.

Semi-thin sections (1.5 μm) were cut with a diamond knife and stained lightly with 1% toluidine blue and examined by light microscopy (Olympus BX51 microscope, Olympus Imaging Corporation, Tokyo, Japan). Later, ultrathin (0.05 μm) sections were cut with a diamond knife (Leica UC6), collected on Formvar-coated single-slot grids, counterstained with 1% uranyl acetate and with Reynold's lead citrate for 10 min. and examined under a FEI Tecnai G2 Spirit TEM. The images were captured with Advanced Microscopy Techniques, using Corp.'s Charge-Coupled Device (CCD from Danvers, MA, USA) imaging system.

The study was carried out with serial ultrathin sections of different enteric ganglia. We checked the entire thickness of each neuron to locate the presence of a basal body or ciliary structure, enabling us to perform a careful reconstruction of the cilium.

## Results

**Fig. 1:**
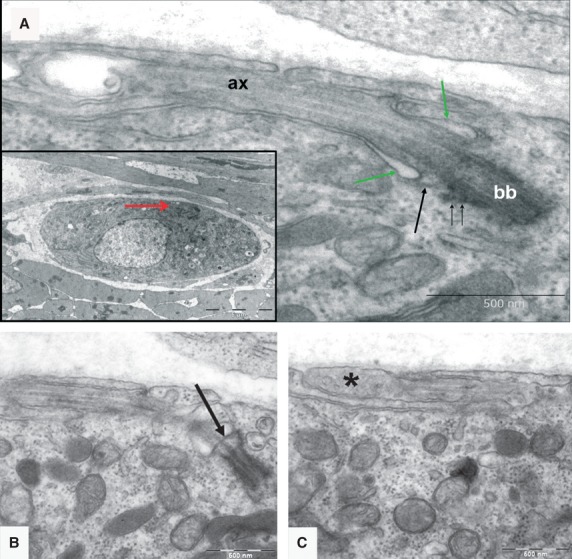
Serial electron micrographs selected from a reconstruction of the primary cilium located in a neuron of the myenteric plexus of rat duodenum. (**A**) The square shows the location of a primary cilium. It emerges from a basal body (bb). Alar sheets (black arrow) extend outwards and contact with plasma membrane. Ciliary pocket (green arrows), where the axoneme (ax) is located. Subdistal appendages (double arrow). (**B**) Terminal plate: opaque structure in the centriolar lumen (arrow). The shaft of the cilia bends at its basis. (**C**) The distal region of the axoneme ends in a bulbous tip (asterisk).

Myenteric ganglia were located intramuscularly in the connective tissue between the circular and longitudinal muscle layers of the duodenum. The ganglion is surrounded by cytoplasmic prolongations of interstitial cells of Cajal (square in the Fig 1A) that constitute a cellular network. We randomly selected different enteric ganglia and systematically studied the serial ultrathin sections made throughout the thickness of each neuron.

The neurons are mainly located in the periphery of the ganglion, surrounded by a large neuropil. The neurons show a large nucleus with uniformly distributed euchromatin and a thin frame of marginal heterocromatin. The square in the Figure 1A shows a primary cilium projected into the extracellular space on the surface of the neuron. The shaft of the cilium appears to bend sharply at its base and lies parallel to the surface of the neuronal membrane. Primary cilia have a length of about 2–4 μm. (Fig. 1A). The axoneme is a direct extension of a typical basal body and contains nine outer doublet microtubules (Fig. 1A–C). It exhibits a 9+0 pattern because the central pair of microtubules is not observed in any of the serial sections, and it lacks motility-related components such as inner and outer dynein arms and radial spokes. The axoneme is surrounded by a membrane that is continuous with the plasma membrane of the neuron. The proximal region of the cilium is located within an invagination of the plasma membrane, the ciliary pocket (Fig. 1A and B).

The distal end of the basal body makes contact with the plasma membrane through transitional fibres (alar sheets; Fig. 1B).

The region separating the basal body and the cilium proper, the transition zone, contains a moderately opaque structure: the terminal plate. This point of contact defines the boundary between the plasma membrane and the ciliary membrane (Fig. 1B).

The diameter of the distal region of the axoneme tends to narrow owing to the centralization and termination of microtubules to form a slightly bulbous tip (Fig. 1C).

**Fig. 2:**
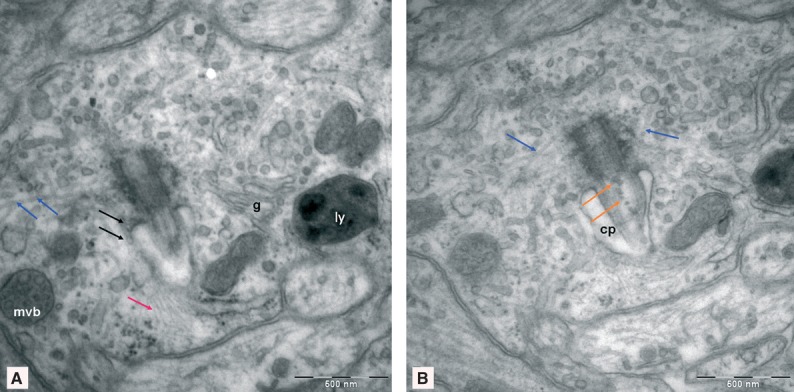
Ciliated neuron with longitudinally sectioned basal body growing a cilium at the neuronal surface. (**A**) Note the presence of several pinocytotic vesicles in the ciliary pocket (black arrow). Microtubules that extend across the neuronal cytoplasm leave from a pericentriolar satellite (blue arrows). In relation to ciliary pocket membrane, microfilaments can be observed (red arrow). g: Golgi cisterns; ly: lysosome; mvb: multivesicular body. (**B**) Vesicular traffic within the basal body and ciliary lumen (orange arrows). cp: ciliary pocket.

Some TEM images indicate that the cilium can remain partially intracellular within a membrane invagination. Small pinocytotic vesicle formation occurs in the basal domain of the ciliary pocket (Fig. 2A). The cilium sheath maintains a connection with the neuronal membrane through bundles of thin filaments whose diameters are compatible with actin filaments. Cytoplasmic microtubules are associated with the satellite basal body from which the primary cilium emerges. As it is shown in the Figure 2A, it results frequent to observe dictyosomes surrounded by multiple secretory vesicles next to the ciliary basal body. This observation suggests an active ciliary transport likely through IFT. In fact, some of these vesicles appear circulating along the lumen of the cilium (Fig. 2B).

**Fig. 3:**
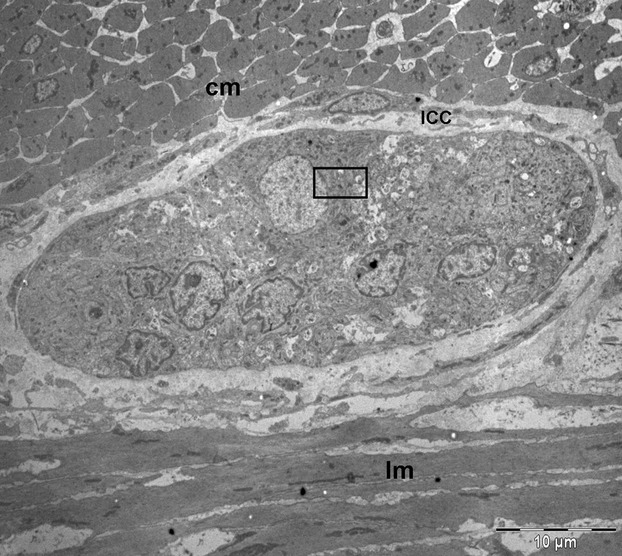
Location of the ciliated neuron in a ganglion of rat duodenum (inset). ICC: interstitial cell of Cajal; lm: longitudinal muscle layer; cm: circular muscle layer.

**Fig. 4:**
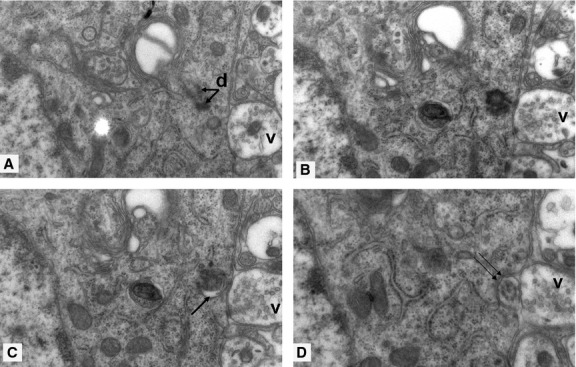
Serial sections of a cilium cut transversely emerging towards the neuronal surface. (**A**) Diplosome (d). One of the centrioles (arrow) will form the basal body of the cilium. (**B**) The basal body of cilium shows radial satellites. (**C**) The cilium is localized deep in the neuronal cytoplasm within an invagination of the plasma membrane (arrow). Cross-section at the level of the basal plate. (**D**) The cilium emerges to the neuropil and contacts with the neuronal membrane (double arrow) and a varicosity which contains small agranular vesicles.

The common features of a ganglion in myenteric plexus are shown in Figure 3. The location of the primary cilium in a neuron is pointed in the inset. This primary cilium trajectory can be appreciated in the serial sections shown in the Figure 4. Starting from the first section, the pair of centrioles (diplosome) from which the cilium originates is observed (Fig. 4A). In the second, the basal body with peripheral satellites can be seen (Fig. 4B). In the third section, the cilium is localized deep in the neuronal cytoplasm within an invagination of the plasma membrane (Fig. 4C). The fourth cut shows the ciliary axoneme located between the neuronal membrane and a cholinergic varicosity. No synaptic specializations are observed in the ciliary membrane (Fig. 4D).

**Fig. 5:**
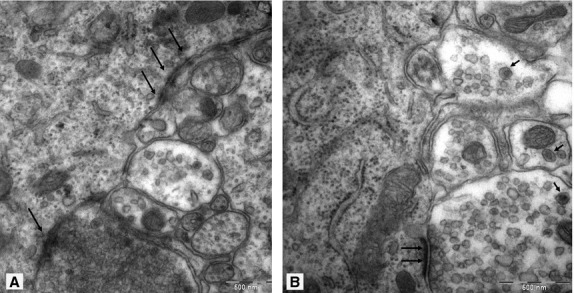
Neuron functionally active. (**A**) Synapses are observed on the surface of the membrane (arrows). (**B**) The cilium establishes contact with an axonal varicosity. Axonal varicosities predominantly contain electron-clear vesicles. Mixed varicosities with large granular vesicles (arrow) and small agranular vesicles, are also observed. At the bottom of the image a synapse of a mixed type can be observed and at the top of the image there is close contact between the ciliary axoneme and varicosities. a: axoneme.

Synapses are recognized by electron dense reinforcements in the post-synaptic neuronal membrane, therefore the ciliated neuron is functionally active (Fig. 5A). Axonal varicosities predominantly contain small agranular vesicles. However, mixed-type varicosities are also frequent, containing both large granular and small agranular vesicles. Varicosities are observed in close contact with the ciliary axoneme, but we could not verify specializations indicative of synapses (Fig. 5B).

## Discussion

Early electron microscopy studies demonstrated evidence of primary cilia in developing and adult neural tissue [18, 23, 45]. Primary cilia are present in neuronal precursor cell populations in the neural tube in the developing embryo [46], and in the post-natal brain in some choroid plexus cells, neural stem cells, neurons and astrocytes within the brain parenchyma [47, 48, 49]. They have also been observed in peripheral neurons in sympathetic ganglia [16] and in Schwann cells in the autonomic nervous system [25]. In these works only a small percentage of electron micrographic sections show primary cilia and it is difficult to identify in detail the components of these neuronal cilia. Likewise it is difficult to appreciate their prevalence without systematic analysis. Some studies reported that neuronal cilia are infrequent, for example in cat spinal cord [50], rat lateral geniculate nucleus [51] and rat ventral tegmental area [52]. Nevertheless, primary cilia are a consistent feature of neurons in the mammalian central nervous system.

Our study was conducted with four different animals, and the enteric ganglia were chosen at random. Although it is difficult to ascertain the number of cilia per cell, careful ultrastructure serial studies carried out on numerous whole neural cellular volumes lead us to the conclusion that there is invariably only one per cell. Given the high frequency of occurrence of primary cilia in neurons, we can consider that the primary cilium is a prevalent nanostructure in neurons of the enteric ganglia of the rat duodenum.

In our study, one primary cilium appears per neuron and its length ranges from 2 to 4 μm. The mean neuronal cilium length varied across brain regions, ranging from 2.1 to 9.4 μm in all the 23 regions of the central nervous system studied [47].

The ultrastructural features that we have defined in our results are consistent with those described in primary cilia present in CNS neurons [29], as well as in astrocytes in the rat CNS [3] and in Schwann cells in the rat PNS [25]. These primary cilia are non-motile cilia derived from the parental centriole of the diplosomal pair. They lack motility-related components such as inner and outer dynein arms, radial spokes and a central pair of microtubules, which are critical for motile cilium [53, 54]. As in many of these descriptions, a large fraction of the mature cilium is localized deep in the cytoplasm within an invagination of the plasma membrane, the ciliary pocket, which has been defined as an endocytic domain for endocytosis by clathrin-coated vesicle formation [55, 56].

Two main ciliogenesis pathways have been reported for primary cilia [57, 58]. In epithelial cells, the mother centriole appears to dock directly at the apical plasma membrane from where the 9+0 axoneme grows towards the extracellular milieu. By contrast, the cilium of fibroblast or smooth muscle cells [59] appears to grow within the cell body, as in the ciliated neurons of the myenteric plexus. Ciliogenesis in enteric neurons involves docking of post-Golgi to the distal end of the mother centriole, emergence of a ciliary bud within the lumen of the vesicle and extension of the axoneme by intraflagellar transport (IFT) [60]; growth of the axoneme takes place within this vesicle, which stretches to form the ciliary shaft and sheath. Fusion of this vesicle with the plasma membrane results in the emergence of the cilium. This process is also similar to those present in interstitial cells of Cajal (ICC) in the enteric plexus of the rat duodenum [61, 62]. This single cilium has been also cited by other authors in mouse gastric ICCs and ICC-like cells in rat mesentery [63, 64].

The primary cilia enclosed in deep invaginations or extending superficially towards the extracellular space has been also reported by other authors [19]. Wheatley [65] proposed that the varying ciliary position is actively regulated by the cell. We do not know the functional significance of the location of the basal body from which the primary cilium emerges (deep or superficial), but obviously this is not a casual arrangement as part of the cilium is protected by the cell membrane (ciliary pocket) forming a specific endocytosis domain [55, 56]. The proximity between the ciliary membrane and the neuronal plasmatic membrane in the ciliary pocket might represent a special area of autocrine signal reception, whereas exposure of the entire surface of the ciliary membrane into the extracellular medium (without forming the ciliary pocket) might imply a more extensive area of reception of paracrine or extracellular matrix signalling, depending on the cell type.

The IFT process is also involved in enteric neuronal cilia maintenance, disassembly and signalling [8]. Intraflagellar transport implies continuous traffic of vesicles from the Golgi apparatus into the cilium, as shown in the images presented in our results.

As there are no descriptions of the presence of single cilia in neurons of the ENS, a study of the cilium function in CNS cells could give us a clue to the possible role of cilia in the ENS cells.

Many regions with particularly long cilia were near brain ventricles. These results suggest that neuronal cilia may detect gradients of peptides and other substances that originate in the cerebrospinal fluid and are conveyed by the extracellular milieu [47]. The primary cilia of enteric neurons project into the neuropil. The ciliary membrane appears to be in contact with the axonal varicosities or the connective tissue surrounding the ganglia and might represent a target for several stimuli.

In the post-natal brain, cilia-mediated Shh signalling is required in neurogenesis for the expansion and establishment of neural precursor cells in the post-natal hippocampus [41], cerebellum [40, 33, 66] and subventricular zone [35–37]. The adult neural stem cells of the dentate gyrus are themselves ciliated [41].

Mutations in ciliary proteins such as CEP290, ARL13B or INPP5E in Joubert syndrome may lead to important neurological disturbances in cerebellar development and defective formation of adult neuronal stem cells regions [45, 49]. These data evidence the relevance of the primary cilium in the CNS neurogenesis.

Could the primary cilium also play a similar role in ENS neurogenesis? In a study about myenteric regeneration after experimental denervation, authors describe the presence of a cilium in some small cells with poorly differentiated cytoplasm found in the ganglia in the reinnervated zone [67]. However, the primary cilium exclusively appears in functionally active neurons in this study.

On the other hand, different receptors such as somatostatin receptor type 3 [27, 68] or serotonin receptor 6 (5-HT_6_) [29], dopamine receptor [31, 32] and p75 neurotrophin receptor (p75^NTR^) [69] selectively localizes to neuronal cilia throughout the rat brain. It can affect many physiological processes in the brain, such as motor activity and cognitive function [27, 68]. This exceptionally high density of receptors on the primary cilium membrane represents an extrasynaptic signalling mechanism [30].

A fundamental understanding of this highly dynamic organelle in the neurons of the enteric nervous system and identification of the consequences of its dysfunction will require the study of cilia in a wide variety of settings.
